# The Therapeutic Relationship in EMDR Therapy

**DOI:** 10.3389/fpsyg.2022.835470

**Published:** 2022-05-31

**Authors:** Michael Hase, Karl Heinz Brisch

**Affiliations:** ^1^EMDR Center, Lüneburg, Germany; ^2^Institute for Early Life Care, Paracelsus Medical University, Salzburg, Austria; ^3^Dr. von Hauner Children's Hospital, University Munich, Munich, Germany

**Keywords:** EMDR therapy, adaptive information processing, therapeutic relationship, attachment theory, consultation, training, research

## Abstract

The history of EMDR Therapy goes back to 1987, when it was introduced as EMD, a novel treatment for PTSD by Francine Shapiro. Over the course of time EMD developed into the comprehensive therapy approach named EMDR Therapy. The development of the “Adaptive Information Processing (AIP) Model”, the model of pathogenesis and change of EMDR Therapy, was a milestone in this development from technique to psychotherapy approach. Up to date EMDR Therapy offers not only a model of pathogenesis and change, but also a variety of treatment plans and techniques to treat patients of various diagnosis far beyond PTSD. What seems to be missing is a specific description of the therapeutic relationship in EMDR Therapy. The therapeutic relationship should be described as a core element of EMDR Therapy, and seems be related to the structure of EMDR Therapy. As attachment theory offers a view on the development of interpersonal relationships in general, an attachment theory based perspective of the therapeutic relationship seems advisable. A description of the therapeutic relationship in EMDR Therapy is necessary at this point of the development of EMDR Therapy to a psychotherapeutic approach and therefore we try to describe the therapeutic relationship in this article and point out parallels between the therapeutic relationship and the development and core features of an attachment based relationship. We propose to describe EMDR Therapy as a sensitive psychotherapy. Implications for treatment, training and research will be discussed.

## Introduction

Eye Movement Desensitization and Reprocessing Therapy (EMDR) consists of a structured set of treatment plans and procedures based on the Adaptive Information Processing (AIP) model (Shapiro and Laliotis, 2011). EMDR was introduced as EMD in 1987 (Shapiro, 1989) as a treatment for PTSD and was developed into the comprehensive therapy approach named EMDR Therapy over the following years. Shapiro intended EMDR Therapy to be compatible with all major orientations of psychotherapy.

Even if treatment plans, named protocols in EMDR Therapy, for very different disorders are available today and different techniques for modification of memory storage are available (Hase, 2021b) the processing of inadequately processed and maladaptively stored memories remains the core of EMDR Therapy. Valiente-Gómez et al. (2017) gave an overview on the application of EMDR Therapy beyond PTSD. Some reflections on the AIP model and theory of pathogenic memories contribute to the theoretical foundation for the evolution of EMDR Therapy (Hase et al., 2017). An overview on the research on working mechanism in EMDR Therapy was recently provided by Landin-Romero et al. (2018). The neurophysiology behind bilateral stimulation has been researched to great extent (Pagani et al., 2017).

The theory currently used to explain EMDR Therapy treatment effects is called the Adaptive Information Processing (AIP) model. The AIP model was developed to explain the rapid change toward positive resolution that can be seen in the EMDR memory reprocessing (Shapiro, 2001). The AIP model assumes “an inherent system in all of us that is physiologically geared to process information to a state of mental health” (Shapiro, 2002). The term information as used in EMDR Therapy refers to affect, cognition, sensory, somatosensory or other internal or external input as perceived at the time of the event leading to memory formation. In EMDR Therapy it is presumed, that the activity of the adaptive information processing system leads to the integration of dysfunctionally encoded information toward functional encoding and adaptive state of memory, in consequence contributing to reduction in distress and/or negative emotions. The AIP system may be hindered or blocked by trauma, other severe stress, or other like the influence of psychoactive drugs in consequence leading to formation of the maldaptively stored memory, which is assumed to be foundational for psychopathology.

In contrast to a common, but nevertheless limited perception of EMDR Therapy, the AIP Model is not just a model of the unprocessed memories, but also of positive, adaptive information, often addressed as “resource”. Shapiro (2001) stressed the fact, that the client would need sufficient resource memory networks, which are present and accessible, for successful memory reprocessing. Shapiro explicitly refers to neglect and abuse in childhood, developmental windows, which might have closed before important infrastructures were set in place, as well object constancy as prerequisites for memory reprocessing. Shapiro states “Once such positive interactions are forged within the therapeutic relationship, they too become stored in memory and can be enhanced through the EMDR procedures.” (Shapiro, 2001, p. 5). In the 3rd edition Shapiro (2018) again addresses these issues, but a bit differently in a case example of a rape victim: “Clinicians must understand how to prepare clients appropriately and stay attuned to to their individual needs while keeping the information processing system activated so learning can take place. Clinicians must take a comprehensive history to identify the appropriate targets for processing and the developmental deficits to be addressed” (Shapiro, 2018, p. 3). Shapiro was even more precise in the 2017 edition of the EMDR Institute Basic Training Manual regarding the necessity of present and accessible adaptive memory networks and referred to the therapeutic relationship as a part of the adaptive memory network: “Adaptive memory networks need to be present and accessible for reprocessing to occur. Therapeutic relationship is part of an adaptive memory network.” (Shapiro and Laliotis, 2017, p. 13).

Interestingly Shapiro mentioned the therapeutic relationship in her textbook, even more explicitly in the EMDR Basic Training Manual, and advised on therapist behavior to be “optimally interactive” (Shapiro, 2007, p. 76), but refrained from describing the therapeutic relationship in EMDR Therapy or on how to establish a secure therapeutic relationship prior to memory reprocessing in detail. As the therapeutic relationship is of great importance regarding the outcome in psychotherapy (Orlinsky et al., 1994) this issue needs to be addressed. The therapeutic relationship is in our view a core component of EMDR Therapy, as EMDR Therapy is definitely psychotherapy. But the therapeutic relationship in EMDR Therapy seems to differ from the therapeutic relationship in other psychotherapeutic approaches.

Dworkin (2005) introduced the relational perspective. Together with Errebo Dworkin brought up important aspects on the issue of the therapeutic process in EMDR Therapy. Dworkin and Errebo (2010), referring to Wachtel (2002), advocate to teach and practice EMDR Therapy as a two-person therapy. This in in our view absolutely necessary as EMDR Therapy is a psychotherapy and the dynamic interplay between therapist and patient is the space, where psychotherapeutic change takes place. In so far a view on other concepts in psychotherapy suggest itself. Piedfort-Marin (2018) relates to the concepts of transference and countertransference, recent considerations on attachment theory for patients with disorganized attachment and how to integrate them in the Adaptive Information Processing (AIP) model. In this extremely valuable article Piedfort-Marin focuses on how the client's and the therapist's conscious and unconscious processes are intertwined and how they may affect the efficacy of EMDR therapy. The integration of the concept of transference and countertransference in the AIP Model is in our view an important contribution. Piedfort-Marin, relating to Dworkin (2005) proposes a definition of transference and countertransference close to the AIP terminology. He describes transference as the activation of dysfunctionally stored (mainly trauma or problematic attachment) memories of the client in relation to the therapy, the clinician, or the relation to the clinician. According to Piedfort-Marin countertransference should be defined as the activation of dysfunctionally stored (mainly trauma or problematic attachment) memories of the therapist by the client, his history, his material, and his relation to the clinician, consciously or unconsciously. We are inline with his emphasis on the importance of the subsystems of social engagement, exploration and cooperation in EMDR Therapy and the importance of an attachment related view in EMDR Therapy. Of course this could lead to bringing up interweaves following a certain train of thought, e.g., transference/countertranference based (Piedfort-Marin, 2018) or relational based (Dworkin, 2005).

Dworkin and Errebo (2010) refer to the relational theory and Stern (2004) ideas of the now moment and the concept of rupture and return. Though is certainly interesting and may have potential for EMDR Therapy sessions, it seems not necessary to leave the AIP Model of EMDR Therapy. On can assume that in EMDR Therapy sessions the AIP System of patient and therapist alike need to be activated and there will be a dynamic interplay, or in other words a reciprocal activation of nodes in therapist and patients memory-networks, leading to patterns of perception and behavior, with positive or negative effects on the course of treatment. This would include also a definition of transference and countertransference in relation to the AIP Model, but is more open or broader as the proposal brought forward by Piedfort-Marin (2018). Such AIP based understanding of clinical phenomena could also integrate the concept of action systems and subsystems (Van der Hart et al., 2006). Dworkin and Errebo (2010) assume, that the risk of potential for relationship rupture in EMDR treatment is very high, referring to the structured approach in EMDR Therapy and the unpredictability of the associative properties of memory reprocessing. In our view we would not overestimate such a risk. The structure of EMDR Therapy safeguards against a high risk of relationship rupture and the attachment perspective may be helpful to understand the properties in EMDR Therapy to develop a strong enough therapeutic relationship as the basis for a profound therapeutic process.

Dworkin and Piedfort-Mari contributed greatly to theory and practice in EMDR Therapy. But a description of the special therapeutic relationship in EMDR Therapy is still missing in the literature. This will be the main objective of this article.

## The Therapeutic Relationship in Psychotherapy

Weisz (1998) uses the terms therapeutic relationship and working alliance equally: “the therapeutic relationship, or working alliance”, whereas otherwise authors distinguish between therapeutic relationship and working alliance. An extensive discussion would be beyond the scope of this article. We consider the therapeutic relationship as basis of the working alliance. There is a broad agreement on the importance of the therapeutic relationship and being considered an integral part of psychotherapy. However, due to very diverging theoretical foundations the definition of the therapeutic relationship differs between the major psychotherapeutic approaches. There have been some efforts to define common elements. One view is that the therapeutic relationship consist of two interrelated parts (Weisz, 1998): the client's positive emotional connection to the therapist, and a shared conceptualization between the client and therapist of the tasks and goals of therapy (Bordin, 1979). Referring to the literature dealing with treatments of adult patients, the development of a positive therapeutic relationship has emerged as a particularly significant process correlated to positive outcome in several studies (Horowitz et al., 1984; Luborsky et al., 1988; Orlinsky et al., 1994). Shirk and Saiz (1992) have argued that this process variable may be an even more significant contributor to outcome for children due to the non-verbal nature of many forms of client-centered and play therapy for children. This seems to be of interest regarding the non-verbal properties of EMDR Therapy. Even if the general importance of the therapeutic relationship in psychotherapy is hardly questioned, it seems quite understandable, that the focus on the psychotherapeutic relationship differs between distinct psychotherapeutic approaches relating to their different theoretical underpinnings. Looking into EMDR Therapy we will certainly recognize strong similarities between EMDR Phase 1 and 2 and “a shared conceptualization between the client and therapist of the tasks and goals of therapy”. One would acknowledge, that a “client's positive emotional connection to the therapist” is of advantage, and vice versa.

Another important topic could be the “idea of man” in EMDR Therapy. The AIP Model implies, that the human being has the innate property of processing information toward an adaptive resolution including appropriate encoding of the memory, leading to symptom reduction as well as personal growth. The “idea of man” in EMDR Therapy is in general the idea of our clients as resourced beings, able to process, to cope with life. Shapiro regards the information processing system as “innate”. Even if we consider this system genetically based we would assume that sensitive interaction between caretaker and baby in the early phases of life are crucial for the development of the adaptive information processing system to full working condition. In addition we have to keep in mind, even if the information processing system is established and active, the capacity for processing would also depend on present and accessible adaptive memories (Shapiro and Laliotis, 2017; Hase, 2021a). The lack of adaptive memories in the early traumatized and neglected clients would not only explain their vulnerability, but also the difficulty in memory reprocessing within therapy.

In general, the pathologic condition refers to blockage of the adaptive information processing system. The therapist acts to facilitate the restart of the adaptive information processing system, keeping it dynamic, in order for reprocessing. In a way the therapist in EMDR Therapy is an expert regarding the structure of the therapeutic process and the felt emotional security in the therapeutic relationship during the memory processing, but not regarding the content of memory reprocessing. This gives the client freedom for the content, enabling the client to engage and stay in his very individual process. These reflections on the “idea of man” in EMDR Therapy seem important, as they determine the supportive stance of the therapist, who will refrain from injecting information into the reprocessing if this is not absolutely necessary. Of course, with the severly traumatized client with attachment deficits or disorders the therapist needs to co-regulate more actively and the formation of a secure bonding within the therapeutic relationship is fundamental. But we still feel the need to describe the therapeutic relationship in EMDR Therapy in greater detail.

## Something to Be Learned From Research and Practice?

Data on the efficacy of EMDR Therapy or on working mechanism in EMDR Therapy are numerous, but in contrast, papers on the therapeutic relationship are rare. The method of qualitative analysis could be helpful in an effort to understand the nature of the therapeutic relationship. Marich published a study on the experience of EMDR Therapy with addicted clients (Marich, 2010) in addiction continuing care. Ten women in an extended-care treatment facility participated in semi-standardized interviews to share their experiences with active addiction, treatment, EMDR therapy, and recovery. Using a descriptive phenomenological psychological method for analysis, four major thematic areas emerged from the interview data: the existence of safety as an essential crucible of the EMDR experience, the importance of accessing the emotional core as vital to the recovery experience, the role of perspective shift in lifestyle change, and the use of a combination of factors for successful treatment. All 10 women, to some degree, credited EMDR treatment as a crucial component of their addiction continuing-care processes, especially in helping with emotional core access and perspective shift. It is to highlight, that these clients felt safe in the EMDR processes even if getting in contact with their emotional core. An unpublished thesis adds to Marichs conclusions (Günther-Soest, 2002). The author used semi-standardized interviews on six traumatized clients which received EMDR Therapy. The clients, five female, one male, had been treated in an in-patient treatment program at a psychiatric hospital in the north of Germany. The semi-standardized interviews were transcribed and analyzed using a descriptive phenomenological psychological method (Langer, 2000). The data revealed that the clients experienced the therapeutic relationship as respectful, paying attention to theirs needs, which determined the direction of their healing process. The clients experienced the therapist respectful stance regarding them as an individual, paying attention to their strength and acknowledging their self-determination as extremely important. They also reported the eye movements being extremely important for the healing process. They reported that the therapeutic relationship had been extremely important, especially in the early phases of their therapy.

Reports from EMDR trained therapist in clinical supervision give the impression, that the therapeutic relationship in EMDR Therapy develops quite fast and seems to be robust with a variety of clients with different backgrounds, from infant to the elderly client, and also with the intellectually disabled client. This seems to be the case even if we take into account the therapist's primary therapeutic approach, before being educated in EMDR Therapy. It seems to be more important that the therapist can bring up an AIP guided understanding of the clients problems and develop an AIP guided treatment plan. Clients reports seem to indicate, that an AIP guided approach in history taking gives the client the impression of a therapist being interested to understand connections between current problems and the biographical background, in a way going for the roots of the clients problems. A significant factor seeming to explain difficulties in the development of the therapeutic relationship and a straightforward or more slow, complicated approach in the course of an EMDR Therapy seems to be the attachment history and attachment status of the client. As promising data on the properties of EMDR Therapy to change the adult attachment status have been provided by Civilotti et al. (2019) and the therapeutic relationship in general is on attachment between client and therapist, a look into attachment theory might lead to some insights.

## Attachment Theory

Attachment Theory refers to Bowlbys foundational observations on small children separated from their primary caretakers from 1958 onwards (Bowlby, 1960) and was published in the trilogy “Attachment and Loss”. Ainsworth developed a theory of a number of attachment patterns or “styles” in infants (Ainsworth et al., 1978). But peer-relationship in all ages and responses to the care needs of adults may be viewed as including some components of attachment behavior (Brisch, 2002; Bowlby, 2005).

According to Brisch (2002) the development of attachment between the infant and caregiver is related to the sensitivity of the caregiver, thus positioning the concept of sensitivity at a pivotal point. As sensitivity facilitates the development of attachment it could play an important role also in the development of a therapeutic relationship (Brisch, 2002). One could assume, that sensitivity could be an important part in describing the therapeutic relationship in EMDR Therapy and we will discuss this in more detail.

## The Therapeutic Relationship in EMDR Therapy and Attachment Theory

As said before Shapiro explicitly mentioned the therapeutic relationship in her textbook, articles and even more explicitly in the 2017 edition of the EMDR Basic Training Manual, but refrained from describing the therapeutic relationship in EMDR Therapy in more detail. Dworkin (2005) published a textbook on the “relational imperative” trying to describe the therapeutic relationship in EMDR treatment. The textbook gives an example how a psychodynamic orientation can be adapted to EMDR Therapy and enrich the therapeutic process. Dworkins statement to view EMDR therapy through the relational lens puts the focus on an important aspect, but is somehow limited. Of course the therapeutic relationship is an important part of EMDR Therapy as it is psychotherapy, but it differs from the therapeutic relationship in other psychotherapeutic approaches to great extent. The genuine AIP, respectively, EMDR Therapy aspect is important.

An important distinction could be, that the therapist in EMDR Therapy should consider himself an expert for the structure of the therapy, but not for the content. An important enough aspect, as structure contributes to a feeling of safety, for therapist and client alike. The idea of the therapist being the expert for the structure, but not the content, is reflected in Shapiros conception of the clients own intrinsic information processing system as being able to process information. The therapist is responsible for activating the system and keeping it a dynamic form. Shapiro repeatedly advises the therapist to refrain from injecting anything in the process, ask open questions and in general “stay out of the way”, just adding bilateral stimulation as long as the information is reprocessing. But many videos of EMDR Therapy memory reprocessing sessions show the therapist being active facilitating the reprocessing, to a great extent non-verbally or with very short comments, the so called verbal support in between sets, e.g. “yes”, “continue”, “I am with you here”, “oh yes, that is hard”, comparable to a mother co-regulating a baby coping with a strong affect.

During other phases in EMDR Therapy, e.g., phase 1, phase 2 or 7, the therapist is of course more active. The diagnostic procedures in phase 1, the therapists interest in the origins of the clients problems, seem to contribute to the developing therapeutic relationship. Of course the ability to attune himself to the client and titrate history taking according to the clients affect tolerance is fundamentally important regarding the development of the therapeutic relationship, as a fine-tuned co-regulation of affect by the caregiver to the infant fosters the development of the attachment. In EMDR Therapy the therapist has a very unique option: to use the developing therapeutic relationship as a resource and apply EMDR resource installation on this resource. Such an approach, like the “Instant Resource Installation” procedure, (Hase, 2021a) facilitates the development of the therapeutic relationship.

The appropriate dosage, speed and rhythm of bilateral stimulation within resource installation and memory reprocessing is of great importance. If the therapist is able to monitor the clients process and adjust the BLS to the clients needs, this contributes to the safe environment and the special therapeutic relationship in EMDR Therapy. This could also contribute to the formation and representation of new memories of experiences of empathy by fine attuned co-regulation of affective arousal within the therapeutic relationship, and is related to the interpersonal experience, the dance between client and therapist, in EMDR Therapy. This can be described to certain extent, but has to be experienced. Offering this experience is a core component in the training of the EMDR therapist as well as in preparation of the client.

As in psychotherapy the attachment system is often activated and therapeutic relationship in general is related to attachment, some reflections on attachment could be added. Flores highlighted, that the need for attachment is a lifelong issue and not limited to childhood (Flores, 2013). The infant has to learn to survive in a world he cannot understand. By repeated experiences in relation to the attachment figure the child learns, what to expect from, e.g., mother. These learning establishes implicit rules relating to the issue “how I have to be or what can I do on my part, to stay in contact with you”. Predictability of the attachment figure is a good basis for the development of the infant. One could assume, that the predictability of therapist behavior in the manualized procedures of EMDR Therapy could contribute to the development of the therapeutic relationship in EMDR Therapy.

According to Brisch (2002) the development of attachment between the infant and caregiver is related to the sensitivity of the caregiver. The caregiver with the most sensitive properties will become the primary attachment figure of the infant. Sensitivity facilitates the development of attachment. How could one define sensitivity? Brisch (2002) outlined that sensitivity shows in speech, rhythm, eye contact and touch. Most important the caregiver has to be able to perceive the infants signals while not misinterpreting them, and react immediately and appropriately. This is only possible if the caregiver is emotionally available for the needs, affects and signals of the infant. In EMDR Therapy as therapists we offer speech, but even more important, rhythm. We keep eye contact, while being not intrusive. Sometimes we offer touch. But most important, we perceive our clients signal, be aware not to intrude, while reacting promptly and appropriately, which will certainly facilitate the development of a secure therapeutic relationship. This shows up in the above mentioned ideas and rules, in procedures and protocols within EMDR Therapy. In our opinion EMDR can be described as a “sensitive” psychotherapy approach. It could explain partly the specific therapeutic relationship in EMDR Therapy, which seems to develop rapidly and offer a safe environment, which allows our clients to reprocess, to grow and to get past the past. Table 1 relates principles of sensitive behavior therapeutic to action in EMDR Therapy.

**Table 1:** Sensitivity and EMDR Therapy.

**Sensitive behavior**	**EMDR Therapy**
Speech	Help to elicit cognition; verbal support during set
Rhythm	Bilateral stimulation; timing of sets and breaks
Eye contact	Aware of facial expression; eye movements
Touch	Tactile stimulation
Perception of client	Awareness during BLS
Not misinterpreting	Refraining from comments; stay out of the way‘
Prompt and appropriate	Keep client in stimulation and react promptly to affective stress of client

## Implications for Training, Consultation, and Research

The development of EMDR Therapy from the trauma-resolution technique toward the conception of the AIP model as a model of the unprocessed information, the nodes behind the symptoms, as well the model of the adaptive information, the resources, was not driven by a distinct theory, but was a dynamic process were chance observation, therapeutic experience and theoretical consideration merged. Meanwhile EMDR Therapy offers the AIP Model as the leading model of pathogenesis and change, a broad range of treatment plans for various disorders and a range of procedures for memory reprocessing or modification. But the development from a technique to a psychotherapy approach calls for description of a structure in EMDR Therapy (Hase, 2021b). Describing a structure in EMDR Therapy referring to six levels with a hierarchy, the AIP Model constituting the uppermost level guiding case conceptualization as well as action in EMDR therapy, down to the level of interventions in processing, could contribute to more clarity in teaching as well as research on applying EMDR Therapy, protocols as well as procedures. In addition the current development of EMDR Therapy calls for a description of the therapeutic relationship in EMDR Therapy.

The development from procedure to psychotherapy approach does somehow reflect in the widely used training structure. Still focussing heavily on the core procedure of memory reprocessing, the phases 4 to 6, often leads the novice in difficulties especially with the complex client with attachment deficits or disorders due to early experiences of neglect and violence experienced within the early relationships. This could be avoided if the training would focus in the beginning to a greater extent on establishing the therapeutic relationship and on enhancing resources, if necessary. This would be in line with Shapiro's above mentioned ideas and reflect the widespread use of resource enhancement procedures (Leeds, 2009). In addition the training should integrate basic knowledge on attachment theory, attachment-oriented self-experience and practice in respect to sensitivity training. The ideas proposed by Dworkin and Errebo (2010) and Piedfort-Marin (2018) to enhance the EMDR Therapy basic training by elements on keeping the patient within the window of tolerance and pay attention to rupture and repair are of great worth and could be integrated in an addition to EMDR basic training.

Such a change in EMDR basic training would also reduce a divergence between the practice of clinical EMDR consultation and training, as many consultants often feel the need to stress these aspects in their work. Reflections on the specific nature of the therapeutic relationship in EMDR Therapy should give reason as the trainee needs to experience this quality and probably needs to be trained to develop this sensitivity. This is common practice in attachment based therapy and could be realized in special workshops.

Research on EMDR Therapy outcome has been very important to give proof of the efficacy of EMDR Therapy and establishing it as an evidence based treatment. The evidence in the treatment of PTSD in adults is very clear. But in the treatment of PTSD in children, in acute presentations, in combat related PTSD, in unipolar depression and in pain evidence is grade I or II relating to Sackett (1989) (Matthijssen et al., 2020). Of course we need more data to reach evidence grade 1, e.g., with unipolar depression. And there are other groups of clients to be treated and efficacy to be shown in new areas. The data on the working mechanism in EMDR Therapy, on the component of bilateral stimulation, are numerous and the insight into the brain of mice (Baek et al., 2019) and men (Pagani et al., 2017) are fascinating. Of course there are still questions open and research must continue. Regarding the therapeutic relationship in EMDR Therapy research is needed. The focus of research could be on the experience of client and therapist alike. The development of a questionaire reflecting on the relational experience of the client in EMDR Therapy could be a step forward. In addition research on the therapist's attachment status in relation to his or her biography and to the therapist's enactment of his or her attachment representation during the therapeutic process would be interesting.

We do hope this article may spark off interest, probably leading to research in more systematic way. Nevertheless, we found it necessary to share these thoughts at this point of the development of EMDR Therapy.

## Summary

The dynamic evolution of EMDR Therapy offers many chances to reach out to clients suffering from various symptoms and problems. The AIP Model seems to be ideal to gain understanding of pathogenesis in a non pathologising way and reach out to the suffering human being, offering an approach, which can be tailored to the individual needs. The AIP model is a model of dysfunctionally stored and affectively highly loaded memories, the nodes behind the symptoms, as well as the model of the adaptive information needed for reprocessing, which should be highlighted more in training and consultation. A description of the therapeutic relationship in EMDR Therapy is necessary at this point of the development of EMDR Therapy to a psychotherapeutic approach and therefore we try to describe the therapeutic relationship in this article and point out parallels between the therapeutic relationship and the development and core features of an attachment based relationship. We propose to describe EMDR Therapy as a sensitive psychotherapy. Relating these ideas onto the therapeutic relationship, in our opinion a core element of EMDR Therapy, and integrating important aspects of attachment theory for a deeper understanding of the therapeutic process, the development of the therapeutic relationship in EMDR Therapy might provide ideas for training, case consultation and will hopefully initiate research.

## Data Availability Statement

The original contributions presented in the study are included in the article/supplementary material, further inquiries can be directed to the corresponding author/s.

## Author Contributions

MH contributed on EMDR Therapy. KB contributed on attachment theory and practice. All authors contributed to the article and approved the submitted version.

## Conflict of Interest

MH is offering education in EMDR Therapy. KB is offering education in attachment-based psychotherapy.

## Publisher's Note

All claims expressed in this article are solely those of the authors and do not necessarily represent those of their affiliated organizations, or those of the publisher, the editors and the reviewers. Any product that may be evaluated in this article, or claim that may be made by its manufacturer, is not guaranteed or endorsed by the publisher.
